# Physical and Psychological Effects of Smartphone App–Based Walking Interventions in Community-Dwelling Older Adults: Systematic Review and Behavior Change Technique–Informed Analysis

**DOI:** 10.2196/78042

**Published:** 2026-02-12

**Authors:** Hiroki Abe, Tomohiro Isinuki, Rinako Ono, Naoki Kanaya, Masaya Tanno, Michiyo Hirano

**Affiliations:** 1 Department of Nursing, School of Health Sciences, Sapporo Medical University, South 1 West 17, Chuo-ku, Sapporo, 060-8556, Japan, 81 116112111 ext 29660; 2 Scholarly Communication Center Library, Sapporo Medical University, Sapporo, Japan

**Keywords:** ICT, information and communication technology, smartphone, apps, walking, BCT, behavior change technique, older adults

## Abstract

**Background:**

With the global increase in population aging, promoting walking as a health behavior to maintain and enhance well-being among older adults has become increasingly important. In recent years, advances in information and communication technology and mobile health have supported the development of health interventions delivered through smartphone apps. However, no review to date has included psychological aspects such as motivation or intention to walk, and the behavior change techniques (BCTs) embedded in smartphone apps that effectively promote walking remain unclear.

**Objective:**

This study had 2 primary aims: (1) to evaluate the effects of smartphone app use on walking behavior and intention to walk among community-dwelling older adults and (2) to identify the specific BCTs delivered through these apps that may promote walking behavior and intention effectively.

**Methods:**

Eligible studies were those published in English or Japanese between March 1, 2015, and February 28, 2025, that focused on community-dwelling older adults, implemented smartphone app–based interventions, and reported walking-related outcomes. A systematic search strategy was designed using keywords such as “older,” “smartphone,” and “walking.” Risk of bias was evaluated using the Study Quality Assessment Tools. The features of the apps described in the selected studies were examined to identify the BCTs they used, as categorized by the BCT Taxonomy.

**Results:**

Of the 296 studies initially retrieved, 8 met the inclusion criteria. These studies varied in terms of participant characteristics, intervention duration, app features, and outcomes, and most were pilot studies. While several apps were designed specifically to increase walking, others included features that facilitated social interaction among users. In addition, 2 studies also reported improved motivation to walk. Apps that were associated with statistically significant improvements in walking behavior frequently used BCTs from the following clusters: (1) goals and planning, (2) feedback and monitoring, and (4) shaping knowledge. Notably, 5 BCTs were not incorporated into any of the reviewed apps.

**Conclusions:**

Although smartphone apps have the potential to improve walking behavior and intention among community-dwelling older adults, the current body of evidence remains limited. Apps that deliver walking-related knowledge, facilitate goal setting, and support behavioral monitoring appear especially effective and may strengthen walking behavior and intention in this population.

## Introduction

In 2024, Japan officially became a super-aged nation, leading a global demographic shift [1,2]. In aging societies such as ours, it is imperative to maintain and improve older adults’ health, especially given the growing burden of chronic diseases and the resulting pressure on health care systems [3]. Walking has been shown to reduce mortality among older adults [4-6] and is consequently recommended as a key health behavior [7,8]. Moreover, walking is one of the most widely adopted and preferred forms of physical activity among older adults [9]. Accordingly, it is particularly important to identify and systematize interventions that promote walking as a strategy for maintaining and enhancing health.

Techniques used to support behavior change have been systematically classified in the Behavior Change Technique (BCT) Taxonomy, which provides a useful framework for analyzing and developing health interventions [10]. Details regarding the BCT Taxonomy, comprising 93 individual techniques organized into 16 clusters, are provided in Multimedia Appendix 1 [11]. Previous studies have demonstrated the effectiveness of BCT-based interventions in promoting physical activity among older adults [12]. Moreover, interventions targeting older populations have already been categorized using the BCT Taxonomy, emphasizing its use in designing and implementing behavioral programs [13]. However, to the best of our knowledge, the specific BCTs that effectively promote walking have not yet been systematically investigated. Therefore, analyzing existing intervention studies on walking promotion through the lens of the BCT Taxonomy and systematically extracting effective intervention components as BCTs is warranted.

In recent years, an increasing number of intervention studies have used smartphone apps in studies involving older adults within the framework of mobile health (mHealth) [14]. Previous reviews have focused on outcomes such as sedentary behavior [14] and physical performance [15], yielding comprehensive insights into specific mHealth features effective in promoting walking among older adults [16]. However, few studies have reported on the psychological effects of mHealth use in this population [17], and to the best of our knowledge, no systematic review has addressed psychological outcomes such as the intention to walk. Existing studies have also underscored the need to address this limitation [16]. Furthermore, current research remains insufficient in elucidating the psychological changes and underlying mechanisms associated with interventions targeting older adults [18,19]. Therefore, it is essential to comprehensively organize both the physical and psychological outcomes related to walking and to determine which smartphone app-based interventions effectively enhance walking behavior and intention among older adults. Moreover, the rapid advancement of information and communication technology (ICT), including the integration of large language models and generative artificial intelligence in health care [20], necessitates continuous knowledge updating and reorganization to maintain relevance and applicability.

Building on the above, the aim of this study was 2-fold: first, to clarify the effects of smartphone app use on walking behavior and intention to walk among community-dwelling older adults; and second, to identify the BCTs delivered through these apps that may effectively promote walking behavior and intention. This study addressed the following research questions:

1. Does the use of smartphone apps improve walking behavior and intention to walk among older adults?
2. Which BCTs, incorporated into smartphone apps, are effective in promoting walking among older adults?

## Methods

### Research Design

This study used a systematic review approach to investigate whether the use of smartphone apps promotes walking behavior among community-dwelling older adults. The review protocol was registered with PROSPERO (International Prospective Register of Systematic Reviews; CRD420251007296) before the commencement of the database search. The PRISMA checklist is available in Checklist 1.

### Search Strategy and Inclusion Criteria

Keywords were identified based on the review questions, and a systematic search was conducted across 5 databases in accordance with PRISMA (Preferred Reporting Items for Systematic Reviews and Meta-Analyses) guidelines. The databases were selected for the following reasons: PubMed, a leading database in medical and health sciences, provides extensive coverage and supports searches using Medical Subject Headings. CINAHL (via EBSCOhost) was included due to its specialization in nursing and allied health literature, which is often underrepresented in traditional medical databases. The Cochrane Database of Systematic Reviews and the Cochrane Central Register of Controlled Trials, both accessed via Wiley Online Library, were selected for their focus on systematic reviews and clinical trials. Finally, Ichushi-Web was included for its comprehensive coverage of Japanese-language literature in medicine, nursing, and health sciences.

The search strategy was developed using a combination of Medical Subject Headings and relevant free-text terms. To reflect the rapidly evolving landscape of eHealth and mHealth research, priority was given to studies using smartphone apps. Consistency in search procedures was ensured to accurately capture studies targeting community-dwelling older adults. To enhance the comprehensiveness of the review, no restrictions were applied to study design during the initial screening phase. The search was conducted on March 19, 2025, and the Boolean operators used are summarized in Table 1. The complete list of search terms is provided in Multimedia Appendix 2. Studies were included if they met the following eligibility criteria:

- Studies were peer-reviewed original research articles.
- Participants were older adults. Given that the definition of older adults varies across countries and studies, older adults were defined as individuals aged 65 years or older, or those explicitly referred to as older adults in the original study, to accommodate regional variation (eg, adults aged ≥60 years in China).
- Studies targeting institutionalized or hospitalized older adults were excluded.
- The intervention used smartphone apps to promote walking. This included apps designed specifically to increase walking, as well as apps with other primary purposes that incorporated walking-promotion features.
- No restrictions were placed on the duration of the intervention.
- Studies in which participants used the app in their daily lives were included, whereas studies conducted in controlled laboratory settings, such as step counter accuracy tests, were excluded.
- Studies were required to report changes in walking-related indicators such as step count, walking frequency, walking distance, walking awareness, or achievement of step-based goals. Studies focusing exclusively on physical function tests, such as the Timed Up and Go Test or the 5-meter walk test, were excluded, as this review emphasized habitual walking behavior and associated intentions.
- Eligible study designs included experimental studies, quasi-experimental studies, and randomized controlled trials (RCTs).
- Studies published in English or Japanese between March 1, 2015, and February 28, 2025, were included.

**Table 1.** Search strings used in academic databases.

Database	Search string
PubMed	(“aged”[MeSH^a^ Terms] OR “elderly”[Title/Abstract] OR “older adult*“[Title/Abstract] OR “older people”[Title/Abstract] OR “older person*“[Title/Abstract] OR “older men”[Title/Abstract] OR “older women”[Title/Abstract] OR “old age”[Title/Abstract] OR “senior citizen*“[Title/Abstract]) AND (“independent living”[MeSH Terms] OR “independent living”[Title/Abstract] OR “community dwell*“[Title/Abstract] OR “community living”[Title/Abstract] OR “community setting”[Title/Abstract] OR “aging in place”[Title/Abstract] OR “aging in place”[Title/Abstract] OR “age in place”[Title/Abstract] OR “non-institutionalized”[Title/Abstract] OR “living at home”[Title/Abstract] OR “healthy”[Title/Abstract] OR “rural”[Title/Abstract]) AND (“smartphone”[MeSH Terms] OR “smartphone*“[Title/Abstract]) AND (“mobile applications”[MeSH Terms] OR “application*“[Title/Abstract] OR “app”[Title/Abstract] OR “mHealth”[Title/Abstract] OR “video games”[MeSH Terms] OR “video game*“[Title/Abstract]) AND (“pedometer”[Title/Abstract] OR “accelerometer”[Title/Abstract] OR “step*“[Title/Abstract] OR “stroll”[Title/Abstract] OR “activ*“[Title/Abstract] OR “walking”[MeSH Terms] OR “walk*“[Title/Abstract] OR “gait”[Title/Abstract] OR “gait analysis”[MeSH Terms] OR “exercise”[MeSH Terms] OR “exerci*"[Title/Abstract])
CINAHL	((MH^b^ aged+) OR (TI^c^ elderly OR AB^d^ elderly) OR (TI “older adult*” OR AB “older adult*“) OR (TI “older people” OR AB “older people”) OR (TI “older person*” OR AB “older person*“) OR (TI “older men” OR AB “older men”) OR (TI “older women” OR AB “older women”) OR (TI “old age” OR AB “old age”) OR (TI “senior citizen*” OR AB “senior citizen*“)) AND ((MH “independent living+“) OR (TI “independent living” OR AB “independent living”) OR (TI “community dwell*” OR AB “community dwell*“) OR (TI “community living” OR AB “community living”) OR (TI “community setting” OR AB “community setting”) OR (TI “aging in place” OR AB “aging in place”) OR (TI “aging in place” OR AB “aging in place”) OR (TI “age in place” OR AB “age in place”) OR (TI non-institutionalized OR AB non-institutionalized) OR (TI “living at home” OR AB “living at home”) OR (TI healthy OR AB healthy) OR (TI rural OR AB rural)) AND ((MH smartphone+) OR (TI smartphone* OR AB smartphone*)) AND ((MH “mobile applications+“) OR (TI application* OR AB application*) OR (TI app OR AB app) OR (TI mHealth OR AB mHealth) OR (MH “video games+“) OR (TI “video game*” OR AB “video game*”)) AND ((TI pedometer OR AB pedometer) OR (TI accelerometer OR AB accelerometer) OR (TI step* OR AB step*) OR (TI stroll OR AB stroll) OR (TI activ* OR AB activ*) OR (MH walking+) OR (TI walk* OR AB walk*) OR (TI Gait OR AB Gait) OR (MH “gait analysis+") OR (MH exercise+) OR (TI exerci* OR AB exerci*))
Cochrane Database of Systematic Reviews	(“aged”[MeSH] OR “elderly”[Title/Abstract] OR “older NEXT adult*“[Title/Abstract] OR “older people”[Title/Abstract] OR “older NEXT person*“[Title/Abstract] OR “older men”[Title/Abstract] OR “older women”[Title/Abstract] OR “old age”[Title/Abstract] OR " senior NEXT citizen*“[Title/Abstract]) AND (“independent living”[MeSH] OR “independent living”[Title/Abstract] OR " community NEXT dwell*“[Title/Abstract] OR “community living”[Title/Abstract] OR “community setting”[Title/Abstract] OR “aging in place”[Title/Abstract] OR “aging in place”[Title/Abstract] OR “age in place”[Title/Abstract] OR “non-institutionalized”[Title/Abstract] OR “living at home”[Title/Abstract] OR “healthy”[Title/Abstract] OR “rural”[Title/Abstract]) AND (“smartphone”[MeSH] OR “smartphone*“[Title/Abstract]) AND (“mobile applications”[MeSH] OR “application*“[Title/Abstract] OR “app”[Title/Abstract] OR “mHealth”[Title/Abstract] OR “video games”[MeSH] OR " video NEXT game*“[Title/Abstract]) AND (“pedometer”[Title/Abstract] OR “accelerometer”[Title/Abstract] OR “step*“[Title/Abstract] OR “stroll”[Title/Abstract] OR “activ*“[Title/Abstract] OR “walking”[MeSH] OR “walk*“[Title/Abstract] OR “Gait”[Title/Abstract] OR “gait analysis”[MeSH] OR “exercise”[MeSH] OR “exerci*"[Title/Abstract])
Cochrane Central Register of Controlled Trials	(“aged”[MeSH] OR “elderly”[Title/Abstract] OR “older NEXT adult*“[Title/Abstract] OR “older people”[Title/Abstract] OR “older NEXT person*“[Title/Abstract] OR “older men”[Title/Abstract] OR “older women”[Title/Abstract] OR “old age”[Title/Abstract] OR " senior NEXT citizen*“[Title/Abstract]) AND (“independent living”[MeSH] OR “independent living”[Title/Abstract] OR " community NEXT dwell*“[Title/Abstract] OR “community living”[Title/Abstract] OR “community setting”[Title/Abstract] OR “aging in place”[Title/Abstract] OR “aging in place”[Title/Abstract] OR “age in place”[Title/Abstract] OR “non-institutionalized”[Title/Abstract] OR “living at home”[Title/Abstract] OR “healthy”[Title/Abstract] OR “rural”[Title/Abstract]) AND (Keywords: “smartphone”[MeSH] OR “smartphone*“[Title/Abstract]) AND (“mobile applications”[MeSH] OR “application*“[Title/Abstract] OR “app”[Title/Abstract] OR “mHealth”[Title/Abstract] OR “video games”[MeSH] OR " video NEXT game*“[Title/Abstract]) AND (“pedometer”[Title/Abstract] OR “accelerometer”[Title/Abstract] OR “step*“[Title/Abstract] OR “stroll”[Title/Abstract] OR “activ*“[Title/Abstract] OR “walking”[MeSH] OR “walk*“[Title/Abstract] OR “Gait”[Title/Abstract] OR “gait analysis”[MeSH] OR “exercise”[MeSH] OR “exerci*"[Title/Abstract])
医中誌Web	(高齢者/TH or 高齢者評価/TH or 高齢/TA or 老年/TA or 老齢/TA or 老人/TA) and (自立生活/TH or 自立/TA or 在住/TA or 居住/TA or 住民/TA or 在宅/TA or 健常/TA or 独居/TA or 地域高齢者/TA) and (スマートフォン/TH or スマートフォン/TA or 携帯電話/TH or 携帯電話/TA) and モバイルアプリケーション/TH or アプリ/TA or (@[モバイルアプリケーション]/TH and @[スマートフォン]/TH) and (歩行/TH or 歩行運動/TH or 歩行分析/TH or 歩数/TA or 歩行/TA or 散歩/TA or 万歩計/TA or “歩数計"/TH)

a MeSH: Medical Subject Headings.

b MH: controlled terms.

c TI: title.

d AB: abstract.

### Study Selection

Prior to the study selection, all authors discussed the inclusion and exclusion criteria based on the Population, Intervention, Comparison, and Outcome framework and reached a consensus. Study selection was conducted in 2 stages by three authors (HA, TI, and MH). The initial screening was conducted using Rayyan. Two authors (HA and TI) independently assessed the titles and abstracts according to predefined eligibility criteria. When a disagreement occurred, a third author (MH) served as the adjudicator. During the second stage, the same two authors independently reviewed the full texts of studies identified as eligible, and any conflicts were resolved through discussion with the third author (MH). To ensure consistency in study selection and data extraction, interrater agreement at each stage was assessed using Cohen kappa (κ) statistic [21].

### Data Extraction

The following information was extracted from each of the included study: study identifiers (eg, author names and publication y), sample characteristics (eg, participant demographics, sample size, and health status), intervention duration, smartphone app details, and reported outcomes. All data were systematically organized and presented in tabular form.

### BCT Classification

First, the lead author conducted an in-depth review of all 8 studies and generated a list of app functions and components. The second author subsequently cross-checked this list against the original articles to ensure accuracy. Finally, all authors reviewed the app functions and purposes described in the eligible studies and classified the corresponding BCTs accordingly. Classification was conducted using the BCT Taxonomy v1, which organizes 93 techniques into 16 clusters [11]. To visualize the relationship between the included studies and the identified BCTs, we created charts at the cluster level. In addition, to closely examine the mechanisms of behavior change, we mapped individual app functions to specific BCTs whenever possible. Any discrepancies were resolved through discussion until full consensus was reached.

### Risk of Bias Assessment

To evaluate the risk of bias in the included studies, the National Institutes of Health Study Quality Assessment Tool was used. This tool is designed to assess the quality of observational cohort and cross-sectional studies involving human participants [22]. The tool comprises either 12 or 14 items, depending on the study design. The proportion of items rated “yes” was calculated for each study to determine overall methodological quality and potential risk of bias. Based on these percentages, categorical quality ratings were defined as follows: high (>70%), moderate (70%‐50%), and low (≤49%).

## Results

### Overview

**Figure 1.**
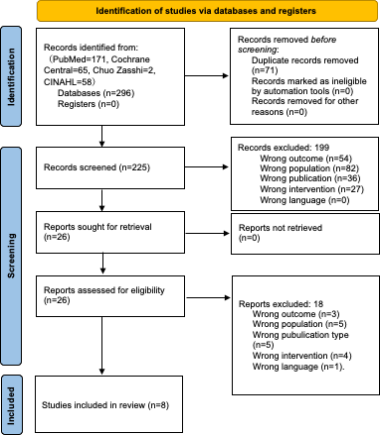
PRISMA (Preferred Reporting Items for Systematic Reviews and Meta-Analyses) flowchart reporting the literature search strategy.

Figure 1 illustrates the screening and selection process for this systematic review. A total of 296 records were identified through 4 database searches: PubMed (n=171), Cochrane Central (n=65), Igaku Chuo Zasshi (n=2), and CINAHL (n=58). A total of 71 duplicate records were collected. During the first screening of titles and abstracts, 4 conflicts between the authors were noted and resolved, and 199 articles were excluded, based on the inclusion criteria. The reasons for exclusion were as follows: incorrect outcomes (n=54), ineligible population (n=82), nonqualifying publication type (n=36), and irrelevant intervention (n=27). In the second screening stage, the full texts of 26 articles were reviewed without any reviewer conflict. Of these, 18 were excluded for the following reasons: incorrect outcome (n=3), ineligible population (n=5), nonqualifying publication type (n=5), irrelevant intervention (n=4), and non-English/Japanese language (n=1), resulting in 8 studies that met all inclusion criteria and were included in the final analysis. During the second screening stage, studies were excluded if the intervention involved smartphone apps for older adults but lacked walking-related outcome measures. For example, studies that assessed broader indicators such as overall physical activity [23] were excluded as having the wrong outcome. An interrater reliability analysis showed a high level of agreement between the authors. During the initial screening, the interrater agreement rate was 98.2%, with a Cohen kappa (κ) of 0.925, indicating almost perfect agreement. In the second screening, agreement reached 100%, with a κ value of 1.0, reflecting perfect concordance.

### Study Characteristics

Table 2 presents an overview of the intervention study outcomes for smartphone apps promoting walking among community-dwelling older adults, along with their associated BCTs. The mean age (SD) of participants in the included studies ranged from 59.8 (6.4) to 78.0 (9.0) years. All participants were ambulatory, although several studies included older adults diagnosed with frailty, prefrailty, or heart failure. The duration of the interventions ranged from 2 weeks (excluding the empirical evaluation study by Zhong and Rau [24]) to 1 year. In terms of study design, none of the included studies used a full RCT. The studies comprised 4 controlled intervention studies, 2 observational cohort or cross-sectional studies, and 2 uncontrolled pre-post studies. The results of the risk-of-bias assessment for each study are presented in Multimedia Appendix 3.

**Table 2.** Overview of intervention study outcomes on smartphone apps promoting walking among community-dwelling older adults and their associated BCT^a^.

Authors	Population and term	Used smartphone app	Findings	Types of BCT^b^	QA^c^
Fong et al [25] (2016)	Older adults capable of walking IG^d^: 97 participants (mean age 60.1, SD 5.5 y) CG^e^: 54 participants (mean age 65.3, SD 8.7 y) 2 wk	Pedometer Lite, Pedometer++, and WalkLogger are existing step-counting apps that enable users to track and manage their daily steps	Willingness to walk (qualitative results)	2	4/14 low
Paul et al [26] (2017)	Healthy older adults: 16 participants (mean 71.1, SD 5.2 y) 6 wk	STARFISH is a smartphone app that promotes physical activity among older adults by tracking steps and providing animated feedback. Users set goals and receive weekly updates. Used in groups of 4, the app fosters motivation through shared progress and group-based rewards	Improving the number of steps	1, 2, 3, 6, 8, 10, and 12	8/12 middle
Matsuoka et al [27] (2024)	Older adults shopping at AEON Malls in Japan (mean 69.9, SD 4.5 y) 1 y	The AEON Mall app, developed for customers of the AEON supermarket chain in Japan, uses GPS to track when users are inside a mall and records their daily step counts. If users exceed 1000 steps in a day, they receive a digital coupon for a lottery to win up to 500 yen (US $3.23) in points. The app encourages physical activity among the general public and may help restore decreased step counts following the COVID-19 pandemic, offering potential benefits for public health.	Improving the number of steps (statistical analysis was not conducted)	2 and 10	10/14 high
Chan et al [28] (2022)	Individuals capable of using a smartphone and walking independently for at least 15 min IG: 70 participants (mean age 59.8, SD 6.4 y) CG: 69 participants (mean age 59.8, SD 6.8 y) 12 wk	ZTExApp is a smartphone app developed to promote physical activity in patients with coronary heart disease by encouraging simple, equipment-free exercises that can be integrated into daily routines. The app offers 4 key features: educational content on ZTEx and its benefits, exercise examples based on 6 fitness components, self-assessment tools like a 30-sec chair stand test, and an electronic diary for goal setting and activity tracking	Improving walking time per week (*P*<.05)	1, 2, and 4	10/14 high
Kawaguchi et al [29] (2024)	This study involved community-dwelling older adults who used smartphones and LINE, spoke Japanese, and were not certified under Japan’s LTCI^f^ system. Participants were divided into an IG (n=85, mean age 69.9) and a CG (n=94, mean age 70.1) 12 wk	The ESP^g^ app is designed to promote social participation among older adults by tracking outing frequency, locations visited, walking distance, and step count using GPS and pedometer data. It classifies users into 4 participation levels—Beginner, Skilled, Master, and Expert—and displays them in a ranking format. The app also provides weekly articles on the importance of social participation. These metrics serve as indicators of both social engagement and daily physical activity.	Improving the number of steps	2, 3, 4, 5, 7, 8, and 10	12/14 high
Ohta et al, [30] (2024)	Thirty older adults 65 y and above who were classified as frail or prefrail 6 wk	Online Kayoinoba is a care prevention app designed to promote physical activity and social interaction among older adults. It features goal setting based on steps and exercise time, municipality-specific exercise videos, meal logging with nutritional balance visualization, social functions like chat and photo sharing, cognitive games to prevent decline, and health check tools. A point system rewards app use, with user activity rankings displayed at national, prefectural, municipal, and community levels.	Improving the number of steps (*P*<.05)	1, 2, 3, 4, 6, 8, and 10	8/12 high
Zhong et al [24] (2020)	Independent, ambulatory older adults (mean age 69.8, SD 7.0 y) Each participant performed 3 types of 40-m indoor walking tests (ST^h^, DT^i^, and FW^j^) only once. Each walking trial was preceded by a 30-second intervention.	Pocket Gait is a smartphone app developed by the authors to evaluate and manage walking quality in the daily lives of older adults. It is designed as a tool to support the early detection of gait abnormalities and fall risk. The app uses the smartphone’s built-in accelerometer to measure 5 gait parameters: step frequency, acceleration RMS^k^, gait regularity, symmetry, and variability. Walking data are collected in real time and presented graphically for user interpretation.	Willingness to walk (qualitative results)	2	6/14 middle
Blomqvist et al [31] (2025)	Physically inactive adults diagnosed with heart failure and equipped with a home-based self-care support device called Optilogg. IG: 10 participants (mean age 78, SD 9 y) CG: 10 participants (mean age 77, SD 5 y) 12 wk	Activity Coach is an mHealth app developed to promote physical activity and reduce sedentary behavior in patients with heart failure. As an extension of the Optilogg self-management platform, it offers features like activity logging in 10-minute intervals, weekly goal setting, educational messages, and visual feedback. Users can define and track their own physical activities, such as walking or housework. The app provides daily educational messages during the first week to build beliefs in the benefits of activity, boost self-efficacy, and emphasize personal goal importance. Progress is displayed visually to support ongoing motivation and behavior change.	Improving the number of steps	1, 2, 1, 5, 7, and 15	6/14 middle

a BCT: behavior change technique.

b The numbers of the types of BCT refer to the following meanings: 1=goals and planning; 2=feedback and monitoring; 3=social support; 4=shaping knowledge; 5=natural consequences; 6=comparison of behavior; 7=associations; 8=repetition and substitution; 9=comparison of outcomes; 10=reward and threat; 11=regulation; 12=antecedents; 13=identity; 14=scheduled consequences; 15=self-belief; and 16=covert learning.

c QA: quality assessment.

d IG: intervention group.

e CG: control group.

f LTCI: long-term care insurance.

g ESP: encouragement of social participation.

h ST: single task.

i DT: dual task.

j FW: fast walk.

k RMS: root mean square.

Among the 6 studies that reported physical outcomes [26-31], 2 demonstrated statistically significant effects of app use on walking behavior [28,30]. Four studies included comparison groups; however, none used a full RCT design. Among the 8 studies, 4 were identified as pilot studies [26,28,30,31] and only 2 of these [28,31] reported predetermined sample sizes sufficient for statistical analysis. Among the 6 studies reporting physical outcomes, 4 conducted statistical analyses [27-29,31]; however, 2 of these did not reach their target sample sizes [29,31].

According to the risk-of-bias assessment, 4 studies were rated as high quality [27-30], 3 as medium quality [24,26,31], and 1 as low quality [25].

### App Features

Of the 8 studies, 1 used a commercially available app, while the remaining 7 used apps developed by the researchers. Six of the 8 apps were specifically designed to promote physical activity by monitoring step counts [24-28,31]. In contrast, 2 apps [29,30] were designed to promote not only physical activity but also social engagement. Six apps were intended for individual use [25-29,31,32], while 2 apps [26,30] incorporated group-based features that allowed users to interact on a shared platform.

Basic step-counting apps such as Pedometer Lite, Pedometer++, and WalkLogger [25] enable users to record and manage daily step counts. STARFISH [26] promotes increased physical activity among older adults by combining step tracking with animated feedback and group-oriented goal setting and reward mechanisms. The AEON Mall app [27] uses GPS to detect user visits to shopping malls and provides digital coupons when users exceed 1000 steps per day, thereby contributing to public health initiatives aimed at restoring physical activity levels that declined following the COVID-19 pandemic.

ZTExApp [28], designed for patients with coronary heart disease, promotes simple, equipment-free exercises and provides educational content, self-assessment tools, and an activity diary. The ESP app [29], aimed at supporting social participation among older adults, records users’ outings, step counts, and visited locations, classifies them into 4 participation levels, and provides weekly educational articles. Online Kayoinoba [30] integrates step goals, exercise videos, dietary tracking, social features, cognitive games, and health checks and uses a point-based system with multilevel rankings to enhance user engagement.

Among the more advanced monitoring tools, Pocket Gait [24] uses smartphone accelerometers to measure walking frequency, root mean square acceleration, regularity, symmetry, and variability, thereby supporting early detection of gait impairments. Activity Coach [31], developed for patients with heart failure, includes activity logs, weekly goal setting, educational messages, and visual feedback to reduce sedentary behavior and strengthen self-efficacy for maintaining physical activity.

### The Effects of Walking Behavior and Intention to Walk by Using a Smartphone App Among Older Adults

Among the included studies, 5 assessed step count as a walking-related outcome, while 1 evaluated weekly walking time. Of these, 1 study reported a statistically significant increase in step count, and another observed a significant improvement in weekly walking time. Outcomes related to intention to walk were reported narratively in 2 studies, both of which indicated positive effects on walking motivation.

### BCT Classification

Table 3 presents the relationship between the apps used in the reviewed studies and the corresponding BCT clusters. Based on the functional features of the apps, the number of BCTs identified per app ranged from 2 to 13 of the 93 techniques defined in the BCT Taxonomy. The smartphone apps incorporated BCTs from 1 to 7 of the 16 clusters defined in the BCT Taxonomy. All apps used techniques from cluster 2: feedback and monitoring. Improvements in walking motivation were also associated with BCTs from cluster 2: feedback and monitoring. Additionally, 4 apps included techniques from clusters 1: goals and planning, and cluster 10: reward and threat. Apps that demonstrated statistically significant effects on walking behavior consistently used techniques from clusters 1, 2, and 4: shaping knowledge. Conversely, 5 BCT clusters were not used in any of the reviewed apps: cluster 9: comparison of outcomes, 11: regulation, 13: identity, 14: scheduled consequences, and 16: covert learning.

**Table 3.** Relationship between apps used by research studies and BCT^a^ clusters^b^.

	Goals and planning	Feedback and monitoring	Social support	Shaping knowledge	Natural consequences	Comparison of behavior	Associations	Repetition and substitution	Comparison of outcomes	Reward and threat	Regulation	Antecedents	Identity	Scheduled consequences	Self-belief	Covert learning
Fong et al [25] (2016)	—^c^	□^d^	—	—	—	—	—	—	—	—	—	—	—	—	—	—
Paul et al [26] (2017)	○^e^	○^e^	○^e^	—	—	○^e^	—	—	—	○^e^	—	○^e^	—	—	—	—
Zhong et al [24] (2020)	—	□^d^	—	—	—	—	—	—	—	—	—	—	—	—	—	—
Chan et al [28] (2022)	●^f^	●^f^	—	●^f^	—	—	—	—	—	—	—	—	—	—	—	—
Matsuoka et al [27] (2024)	—	○^e^	—	—	—	—	—	—	—	○^e^	—	—	—	—	—	—
Ohta et al [30] (2024)	●^f^	●^f^	●^f^	●^f^	—	●^f^	—	●^f^	—	●^f^	—	—	—	—	—	—
Kawaguchi et al [29] (2024)	—	○^e^	○^e^	○^e^	○^e^	—	○^e^	○^e^	—	○^e^	—	—	—	—	—	—
Blomqvist et al [31] (2025)	○^e^	○^e^	—	—	○^e^	—	○^e^	—	—	—	—	—	—	—	○^e^	—
Total number of BCT types^g^	4	8	3	3	2	2	2	2	0	4	0	1	0	0	1	0

a BCT: behavior change technique.

b The above table shows the relationship between each study and the BCT clusters.

c Not applicable.

d The study demonstrated qualitative effects.

e The effect was not statistically significant.

f A statistically significant effect.

g The last row shows the total number of occurrences in each cluster.

## Discussion

### Overview

This systematic review investigated the effectiveness of smartphone apps on walking behavior and the intention to walk among community-dwelling older adults, based on 8 existing studies. Although the number of included studies was smaller than in comparable reviews (eg, Llopis et al [16]), this review distinctively focused on community-dwelling older adults, excluding those residing in institutions or hospitals, and explicitly evaluated both walking behavior and walking intention. Furthermore, by classifying the BCTs embedded in app functions, the review identified app-based intervention components that may effectively promote walking.

### Does the Use of Smartphone Apps Improve Walking Behavior and Intention to Walk Among Older Adults?

The use of smartphone apps demonstrated positive effects on both walking behavior and walking intention [24,25,28,30]. Among the included studies, 6 measured physical outcomes (eg, step count or walking duration), while 2 assessed psychological outcomes such as walking intention. Although both studies reporting psychological outcomes relied on narrative data, indicating the need for further quantitative examination, the findings suggest that smartphone apps may have the potential to improve both physical and psychological outcomes related to walking.

The 2 studies that assessed psychological outcomes used apps that included step-monitoring functions. Previous research has demonstrated that activity tracker use can effectively increase step counts [33], suggesting that enabling older adults to self-monitor their walking behavior may also exert a positive influence on their walking intention.

Most smartphone app-based intervention studies targeting older adults have been pilot studies [34], a trend that is consistent with observations reported in previous systematic reviews [16]. Given this trend and the limited number of eligible studies, the conclusions of this review should be interpreted with caution. Nevertheless, some studies reported statistically significant improvements in walking behavior among community-dwelling older adults following app use, and narrative findings suggested psychological benefits as well [24,25].

### Which BCTs Incorporated Into Smartphone Apps Are Effective in Promoting Walking Among Older Adults?

Among the studies included in this review, 2 smartphone app–based interventions [28,30] demonstrated statistically significant improvements in walking-related outcomes. Both of these apps implemented the following BCT clusters: (1) goal setting and planning, (2) feedback and monitoring, and (3) shaping knowledge. This overlap holds theoretical significance for the design of smartphone apps aimed at promoting walking among older adults. Gilchrist et al [13] demonstrated that interpersonal physical activity programs incorporating goal setting and self-monitoring effectively enhance physical activity levels, which aligns with the findings of this review.

Although the BCT Taxonomy provides a comprehensive framework for behavior change, this review suggests that, in the context of walking promotion among older adults, providing relevant knowledge, facilitating goal setting and planning, and enabling behavior monitoring may be especially effective. It is also important that apps designed for older adults maintain simplicity and minimize cognitive burden [35]. Because the number of implemented BCTs is not necessarily associated with increased physical activity [36], overly complex or multifunctional apps may impose unnecessary burden and hinder sustained use. Therefore, designing apps that focus on the specific BCTs shown to be effective is likely to lead to more desirable outcomes.

According to the BCT Taxonomy, all apps reviewed in this study incorporated cluster 2: feedback and monitoring, and positive effects were observed on both walking behavior and walking intention. These apps provided feedback related to walking behavior, such as step counts and activity levels. Smartphones are highly portable and are well-suited for real-time monitoring of physical activity [37], and health platforms such as Apple’s HealthKit [38] and Android’s Google Fit [39] allow developers to efficiently integrate feedback and monitoring functions via available application programming interfaces [40]. This not only facilitates the implementation of BCT-aligned features but also reduces development costs, offering advantages from both behavioral science and engineering perspectives.

In contrast, 5 of the 16 BCT clusters were not implemented in any of the reviewed apps. The absence of these techniques may reflect technological constraints inherent in app-based interventions. For instance, BCT 13.1 (identification of self as a role model) necessitates a high degree of self-reflection or interaction with professionals, making it challenging to implement in apps structured around predefined conditional logic [40]. Many of the reviewed apps were designed to deliver standardized, rule-based responses to user behavior, thereby limiting their capacity to offer context-sensitive or highly personalized feedback.

According to the scoping review by Panicker et al [41], smartphone apps were the most frequently used tools in interventions using wearable devices to promote physical activity, although wrist-worn and pendant-type devices were also used. Selecting hardware and software that supports user engagement and integrating them when appropriate may facilitate the implementation of a broader range of BCTs. Additionally, an RCT conducted by Lunde et al [42], which was excluded from this review due to population mismatch, reported a significant increase in step count when professional support was delivered via a smartphone app. This finding suggests that integrating smartphone apps with professional guidance may enable more personalized interventions and generate synergistic effects. Furthermore, with the rapid advancement of generative artificial intelligence in health care [20], large language models can now be integrated into mHealth interventions via application programming interfaces, thereby enabling more personalized and adaptive interventions [43]. This development may expand the capacity of mHealth systems to support a broader range of BCTs in the future.

### Limitations

This study has several limitations that warrant caution when interpreting the findings. First, none of the included studies were full RCTs, so the review lacks high-level evidence on the effectiveness of smartphone apps for promoting walking among older adults. Second, many of the reviewed studies featured research designs that may be considered suboptimal, likely reflecting the challenge of keeping pace with the rapid evolution of ICT. This limitation could have resulted in the underestimation of the health effects of ICT-based interventions, potentially biasing the results of this review. Third, the operational definition of “older adults” varied across studies, introducing heterogeneity and potential bias. Moreover, older adults who are able to use ICT tend to be relatively younger and of higher sociodemographic status, reflecting a selection bias. This inherent selection bias should be considered when evaluating the generalizability of our findings. Fourth, this review included only 8 studies, which is fewer than in reviews encompassing older adults with more diverse sociodemographic backgrounds [28]. Because this review focused exclusively on community-dwelling older adults rather than those residing in institutions or hospitals, its findings may not generalize to populations with different backgrounds. Finally, this type of research has inherent limitations that should be acknowledged, such as publication bias (the tendency for statistically significant studies to be published preferentially) [44] and selection bias (the possibility that eligible studies were not captured in the search) [45].

### Conclusion

In this study, we conducted a systematic review of 8 studies examining the effects of smartphone app use on walking behavior among community-dwelling older adults. Several studies reported improvements in step counts and intention to walk. Despite the limited number of eligible studies, the findings suggest that apps incorporating features to support walking-related knowledge acquisition, goal setting and planning, and behavioral monitoring may be especially effective in promoting walking behavior and intention among older adults.

## Supplementary material

10.2196/78042 Multimedia Appendix 1 Details of the behavior change techniques taxonomy.

10.2196/78042 Multimedia Appendix 2 Literature search strategy.

10.2196/78042 Multimedia Appendix 3 Study quality assessment tools.

10.2196/78042 Checklist 1 PRISMA 2020 checklist.

## Abbreviations

BCT: behavior change technique
ICT: information and communication technology
mHealth: mobile health
PRISMA: Preferred Reporting Items for Systematic Reviews and Meta-Analyses
PROSPERO: International Prospective Register of Systematic Reviews
RCT: randomized controlled trial
