# Supplementary material for: Physical and Psychological Effects of Smartphone App–Based Walking Interventions in Community-Dwelling Older Adults: Systematic Review and Behavior Change Technique–Informed Analysis
Source: JMIR Hum Factors. 2026 Feb 12;13:e78042. doi: 10.2196/78042 (PMC12900276; doi:10.2196/78042)
Supplement: Multimedia Appendix 1 [file humanfactors-v13-e78042-s001.docx]

| 16 clusters | 93 techniques |
| --- | --- |
| Goals and planning | Goal setting (behavior), Problem solving, Goal setting (outcome), Action planning, Review behavior goal(s), Discrepancy between current behavior and goal, Review outcome goal(s), Behavioral contract and Commitment |
| Feedback and monitoring | Monitoring of behavior by others without feedback, Feedback on behaviour, Self-monitoring of behaviour, Self-monitoring of outcome(s) of behaviour, Monitoring of outcome(s) of behavior without feedback, Biofeedback and Feedback on outcome(s) of behavior |
| Social support | Social support (unspecified), Social support (practical) and Social support (emotional) |
| Shaping knowledge | Instruction on how to perform the behavior, Information about Antecedents, Re-attribution and Behavioral experiments |
| Natural consequences | Information about health consequences, Salience of consequences, Information about social and environmental consequences, Monitoring of emotional consequences, Anticipated regret and Information about emotional consequences |
| Comparison of behaviour | Demonstration of the behavior, Social comparison and Information about others’ approval |
| Associations | Prompts/cues, Cue signalling reward, Reduce prompts/cues, Remove access to the reward, Remove aversive stimulus, Satiation, Exposure and Associative learning |
| Repetition and substitution | Behavioral practice/rehearsal, Behavior substitution, Habit formation, Habit reversal, Overcorrection, Generalisation of target behavior and Graded tasks |
| Comparison of outcomes | Credible source, Pros and cons and Comparative imagining of future outcomes |
| Reward and threat | Material incentive (behavior), Material reward (behavior), Non-specific reward, Social reward, Social incentive, Non-specific incentive, Self-incentive, Incentive (outcome), Self-reward, Reward (outcome) and Future punishment |
| Regulation | Pharmacological support, Reduce negative emotions, Conserving mental resources and Paradoxical instructions |
| Antecedents | Restructuring the physical environment, Restructuring the social environment, Avoidance/reducing exposure to cues for the behavior, Distraction, Adding objects to the  Environment and Body changes |
| Identity | Identification of self as role model, Framing/reframing, Incompatible beliefs, Valued self-identify, Identity associated with changed behavior |
| Scheduled consequences | Behavior cost, Punishment, Remove reward, Reward approximation, Rewarding completion, Situation-specific reward, Reward incompatible behavior, Reward alternative behavior, Reduce reward frequency and Remove punishment |
| Self-belief | Verbal persuasion about capability, Mental rehearsal of successful performance, Focus on past success, Self-talk |
| Covert learning | Imaginary punishment, Imaginary reward, Vicarious consequences |
