# Supplementary material for: Physical and Psychological Effects of Smartphone App–Based Walking Interventions in Community-Dwelling Older Adults: Systematic Review and Behavior Change Technique–Informed Analysis
Source: JMIR Hum Factors. 2026 Feb 12;13:e78042. doi: 10.2196/78042 (PMC12900276; doi:10.2196/78042)
Supplement: Multimedia Appendix 2 [file humanfactors-v13-e78042-s002.docx]

**Multimedia Appendix 2.** Combination of Medial Subject Headings (MeSH) and text words used for the literature search in different database

The search strategy was organized into five concepts. Within each concept, MeSH terms and text words were combined using OR, and the five concepts were then combined using AND to construct the final search strategy. The detailed terms for each concept are listed below.

**PubMed**

**Concept 1: Older Adults**

**Keywords:** "aged"[MeSH Terms] OR "elderly"[Title/Abstract] OR "older adult*"[Title/Abstract] OR "older people"[Title/Abstract] OR "older person*"[Title/Abstract] OR "older men"[Title/Abstract] OR "older women"[Title/Abstract] OR "old age"[Title/Abstract] OR "senior citizen*"[Title/Abstract]

**Concept 2: Community Dwelling**

**Keywords:** "independent living"[MeSH Terms] OR "independent living"[Title/Abstract] OR "community dwell*"[Title/Abstract] OR "community living"[Title/Abstract] OR "community setting"[Title/Abstract] OR "aging in place"[Title/Abstract] OR "ageing in place"[Title/Abstract] OR "age in place"[Title/Abstract] OR "non-institutionalized"[Title/Abstract] OR "living at home"[Title/Abstract] OR "healthy"[Title/Abstract] OR "rural"[Title/Abstract]

**Concept 3: Smartphone**

**Keywords:** "smartphone"[MeSH Terms] OR "smartphone*"[Title/Abstract]

**Concept 4: Mobile Applications**

**Keywords:** "mobile applications"[MeSH Terms] OR "application*"[Title/Abstract] OR "app"[Title/Abstract] OR "mHealth"[Title/Abstract] OR "video games"[MeSH Terms] OR "video game*"[Title/Abstract]

**Concept 5: Walking**

**Keywords:** "pedometer"[Title/Abstract] OR "accelerometer"[Title/Abstract] OR "step*"[Title/Abstract] OR "stroll"[Title/Abstract] OR "activ*"[Title/Abstract] OR "walking"[MeSH Terms] OR "walk*"[Title/Abstract] OR "Gait"[Title/Abstract] OR "gait analysis"[MeSH Terms] OR "exercise"[MeSH Terms] OR "exerci*"[Title/Abstract]

**CINAHL（EBSCOhost）**

**Concept 1: Older Adults**

**Keywords:** (MH aged+) OR (TI elderly OR AB elderly) OR (TI "older adult*" OR AB "older adult*") OR (TI "older people" OR AB "older people") OR (TI "older person*" OR AB "older person*") OR (TI "older men" OR AB "older men") OR (TI "older women" OR AB "older women") OR (TI "old age" OR AB "old age") OR (TI "senior citizen*" OR AB "senior citizen*")

**Concept 2: Community Dwelling**

**Keywords:** (MH "independent living+") OR (TI "independent living" OR AB "independent living") OR (TI "community dwell*" OR AB "community dwell*") OR (TI "community living" OR AB "community living") OR (TI "community setting" OR AB "community setting") OR (TI "aging in place" OR AB "aging in place") OR (TI "ageing in place" OR AB "ageing in place") OR (TI "age in place" OR AB "age in place") OR (TI non-institutionalized OR AB non-institutionalized) OR (TI "living at home" OR AB "living at home") OR (TI healthy OR AB healthy) OR (TI rural OR AB rural)

**Concept 3: Smartphone**

**Keywords:** (MH smartphone+) OR (TI smartphone* OR AB smartphone*)

**Concept 4: Mobile Applications**

**Keywords:** (MH "mobile applications+") OR (TI application* OR AB application*) OR (TI app OR AB app) OR (TI mHealth OR AB mHealth) OR (MH "video games+") OR (TI "video game*" OR AB "video game*")

**Concept 5: Walking**

**Keywords:** (TI pedometer OR AB pedometer) OR (TI accelerometer OR AB accelerometer) OR (TI step* OR AB step*) OR (TI stroll OR AB stroll) OR (TI activ* OR AB activ*) OR (MH walking+) OR (TI walk* OR AB walk*) OR (TI Gait OR AB Gait) OR (MH "gait analysis+") OR (MH exercise+) OR (TI exerci* OR AB exerci*)

**Cochrane Database of Systematic Reviews（Wiley Online Library）**

**Concept 1: Older Adults**

**Keywords:** "aged"[MeSH] OR "elderly"[Title/Abstract] OR "older NEXT adult*"[Title/Abstract] OR "older people"[Title/Abstract] OR "older NEXT person*"[Title/Abstract] OR "older men"[Title/Abstract] OR "older women"[Title/Abstract] OR "old age"[Title/Abstract] OR " senior NEXT citizen*"[Title/Abstract]

**Concept 2: Community Dwelling**

**Keywords:** "independent living"[MeSH] OR "independent living"[Title/Abstract] OR " community NEXT dwell*"[Title/Abstract] OR "community living"[Title/Abstract] OR "community setting"[Title/Abstract] OR "aging in place"[Title/Abstract] OR "ageing in place"[Title/Abstract] OR "age in place"[Title/Abstract] OR "non-institutionalized"[Title/Abstract] OR "living at home"[Title/Abstract] OR "healthy"[Title/Abstract] OR "rural"[Title/Abstract]

**Concept 3: Smartphone**

**Keywords:** "smartphone"[MeSH] OR "smartphone*"[Title/Abstract]

**Concept 4: Mobile Applications**

**Keywords:** "mobile applications"[MeSH] OR "application*"[Title/Abstract] OR "app"[Title/Abstract] OR "mHealth"[Title/Abstract] OR "video games"[MeSH] OR " video NEXT game*"[Title/Abstract]

**Concept 5: Walking**

**Keywords:** "pedometer"[Title/Abstract] OR "accelerometer"[Title/Abstract] OR "step*"[Title/Abstract] OR "stroll"[Title/Abstract] OR "activ*"[Title/Abstract] OR "walking"[MeSH] OR "walk*"[Title/Abstract] OR "Gait"[Title/Abstract] OR "gait analysis"[MeSH] OR "exercise"[MeSH] OR "exerci*"[Title/Abstract]

**Cochrane Central Register of Controlled Trials（Wiley Online Library）**

**Concept 1: Older Adults**

**Keywords:** "aged"[MeSH] OR "elderly"[Title/Abstract] OR "older NEXT adult*"[Title/Abstract] OR "older people"[Title/Abstract] OR "older NEXT person*"[Title/Abstract] OR "older men"[Title/Abstract] OR "older women"[Title/Abstract] OR "old age"[Title/Abstract] OR " senior NEXT citizen*"[Title/Abstract]

**Concept 2: Community Dwelling**

**Keywords:** "independent living"[MeSH] OR "independent living"[Title/Abstract] OR " community NEXT dwell*"[Title/Abstract] OR "community living"[Title/Abstract] OR "community setting"[Title/Abstract] OR "aging in place"[Title/Abstract] OR "ageing in place"[Title/Abstract] OR "age in place"[Title/Abstract] OR "non-institutionalized"[Title/Abstract] OR "living at home"[Title/Abstract] OR "healthy"[Title/Abstract] OR "rural"[Title/Abstract]

**Concept 3: Smartphone**

**Keywords:** "smartphone"[MeSH] OR "smartphone*"[Title/Abstract]

**Concept 4: Mobile Applications**

**Keywords:** "mobile applications"[MeSH] OR "application*"[Title/Abstract] OR "app"[Title/Abstract] OR "mHealth"[Title/Abstract] OR "video games"[MeSH] OR " video NEXT game*"[Title/Abstract]

**Concept 5: Walking**

**Keywords:** "pedometer"[Title/Abstract] OR "accelerometer"[Title/Abstract] OR "step*"[Title/Abstract] OR "stroll"[Title/Abstract] OR "activ*"[Title/Abstract] OR "walking"[MeSH] OR "walk*"[Title/Abstract] OR "Gait"[Title/Abstract] OR "gait analysis"[MeSH] OR "exercise"[MeSH] OR "exerci*"[Title/Abstract]

**医中誌Web**

**Concept 1: 高齢者**

**Keywords:** 高齢者/TH or 高齢者評価/TH or 高齢/TA or 老年/TA or 老齢/TA or 老人/TA

**Concept 2: 地域在住**

**Keywords:** 自立生活/TH or 自立/TA or 在住/TA or 居住/TA or 住民/TA or 在宅/TA or 健常/TA or 独居/TA or 地域高齢者/TA

**Concept 3: スマートフォン**

**Keywords:** スマートフォン/TH or スマートフォン/TA or 携帯電話/TH or 携帯電話/TA

**Concept 4: アプリケーション**

**Keywords:** モバイルアプリケーション/TH or アプリ/TA or (@[モバイルアプリケーション]/TH and @[スマートフォン]/TH)

**Concept 5: 歩行**

**Keywords:** 歩行/TH or 歩行運動/TH or 歩行分析/TH or 歩数/TA or 歩行/TA or 散歩/TA or 万歩計/TA or "歩数計"/TH
